# Phosphodiesterases Regulate *C. elegans* Bitter Taste Avoidance

**DOI:** 10.17912/micropub.biology.001946

**Published:** 2025-12-04

**Authors:** Savannah E. Sojka, Fletcher M. Hammond, Denise M. Ferkey

**Affiliations:** 1 Department of Biological Sciences, University at Buffalo, State University of New York, Buffalo, New York, United States; 2 Rochester General College of Health Careers, Rochester, New York, United States; 3 Biology Department, Allegheny College, Meadville, Pennsylvania, United States

## Abstract

Cyclic nucleotides (cAMP, cGMP) are critical second messengers that transduce diverse signaling events within cells, ranging from developmental processes and neuronal signaling to roles in cellular growth (Fajardo et al., 2014; Galande & Cote, 2025; Levy et al., 2011). Thus, the regulation of cyclic nucleotide levels is critical for the regulation of signal transduction pathways and cellular homeostasis.&nbsp; We previously reported a role for cGMP in the negative regulation of bitter taste avoidance in *C. elegans* (Chaubey et al., 2023; Krzyzanowski et al., 2013; Krzyzanowski et al., 2016).&nbsp; Phosphodiesterases (PDEs) are a class of enzymes that break down cyclic nucleotides, and we show here that multiple PDEs likely work together to modulate *C. elegans* bitter taste sensitivity.

**Figure 1. Multiple PDEs function together to modulate response to select taste stimuli:**
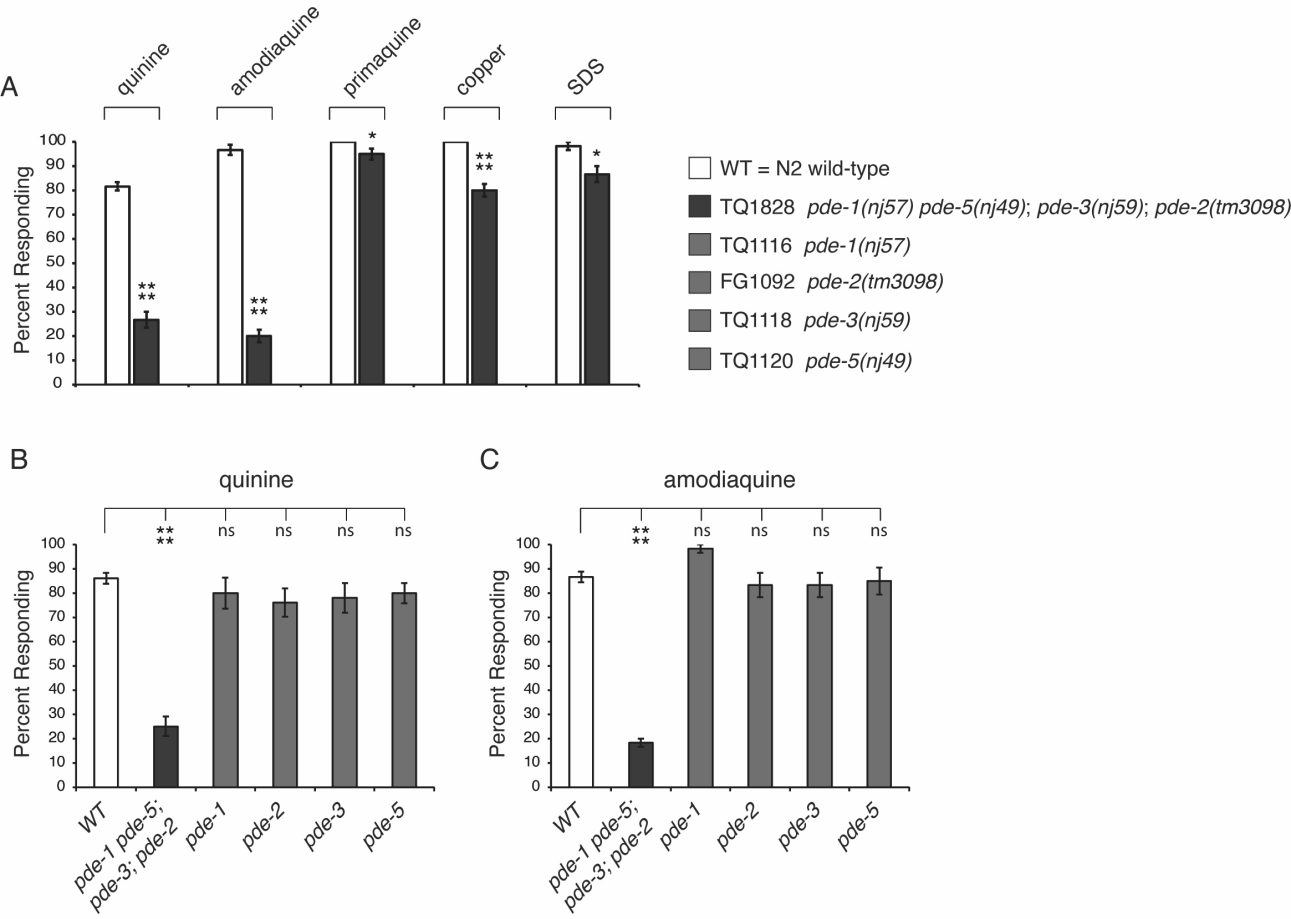
*C. elegans* respond to diverse taste stimuli.&nbsp; (A) Animals lacking the function of four PDEs simultaneously ( PDE-1 , PDE-5 , PDE-3 , PDE-2 ) are severely defective in response to the bitter tastants quinine and amodiaquine. &nbsp;(B-C) While the quadruple mutant is defective in response to quinine (B) and amodiaquine (C), single mutants lacking the function of PDE-1 , PDE-2 , PDE-3 or PDE-5 individually respond similarly to wild-type animals.&nbsp; ns = not significant.&nbsp; The percentage of animals responding is shown.&nbsp; The combined data of ≥ 3 independent trials and ≥ 60 animals is shown in each panel.&nbsp; Concentrations used: 10mM quinine, 10mM amodiaquine, 10mM primaquine, 10mM CuCl _2_ , 0.01% SDS.

## Description

*C. elegans* sensitivity to aversive stimuli is modulated by feeding status, such that animals are more responsive to aversive cues when they are well fed, and less sensitive when starved (Chao et al., 2004; Ezcurra et al., 2011; Ferkey et al., 2007; Harris et al., 2009; Krzyzanowski et al., 2016; Wragg et al., 2007).&nbsp; While biogenic amines play an important role in this regulation, intracellular neuronal cGMP levels also contribute to dampening responses to select ASH-detected stimuli.&nbsp; Previous studies suggested that upon food deprivation, cGMP is produced by the ODR-1 guanylyl cyclase in the AWB, AWC and ASI neurons, and by an unidentified guanylyl cyclase in ADL, and then moves through a modulatory network of neuronal gap junction connections to the ASH nociceptors. Once there, it acts to dampen signaling via activation of the cGMP-dependent protein kinase EGL-4 , which then likely phosphorylates and activates RGS-2 and RGS-3 , which subsequently downregulate G protein-coupled signaling in ASH (Chaubey et al., 2023; Krzyzanowski et al., 2013; Krzyzanowski et al., 2016; Sojka et al., 2025; Voelker et al., 2019). &nbsp;Accordingly, loss of the guanylyl cyclases that produce the modulatory pool of cGMP ( *odr-1* , and also likely *gcy-27* , *gcy-33* , *gcy-34* ), loss of innexins that facilitate its transport through gap junctions ( *inx-4* , *inx-7* , *inx-15* , *inx-16* , *inx-17* , *inx-18* , *inx-19* , *inx-20* , *unc-7* , *unc-9* ), or loss of the regulatory components ( *egl-4* , *rgs-2* , *rgs-3* ) all result in elevated behavioral sensitivity to the bitter tastant quinine (Chaubey et al., 2023; Krzyzanowski et al., 2013; Krzyzanowski et al., 2016; Sojka et al., 2025; Voelker et al., 2019), which is detected primarily by the ASH nociceptors (Hilliard et al., 2004).&nbsp; Conversely, a gain-of-function mutation in EGL-4 (Krzyzanowski et al., 2013), or ectopic cGMP generation (Chaubey et al., 2023; Krzyzanowski et al., 2016), both lead to diminished behavioral sensitivity to quinine.

Six phosphodiesterase (PDE) enzymes are encoded by the *C. elegans* genome (Galande & Cote, 2025).&nbsp; Of these, two ( PDE-4 and PDE-6 ) have high sequence similarity to mammalian PDEs that have a strong preference for cAMP, while the remaining four ( PDE-1 , PDE-2 , PDE-3 , PDE-5 ) may have the dual ability to break down both cAMP and cGMP (Galande & Cote, 2025; Hahm et al., 2009; Lugnier, 2006), making them potential candidates for regulating bitter taste signaling.&nbsp; To determine whether loss-of-function mutations in phosphodiesterase encoding genes impact nociceptive behavior, we assessed responses to five different aversive stimuli.&nbsp; The ASH sensory neurons are the primary detectors of the bitter tastant quinine and possibly other bitter compounds (Hilliard et al., 2004), as well as the heavy metal copper and the detergent SDS that are not thought to signal through G protein-coupled receptors (Hilliard et al., 2005; Hilliard et al., 2002; Sambongi et al., 1999).&nbsp; Quadruple mutant animals [ *pde-1 ( nj57 ) pde-5 ( nj49 )* ; *pde-3 ( nj59 )* ; *pde-2 ( tm3098 )* ] were profoundly defective for response to the bitter tastants quinine and amodiaquine ( Figure 1A ).&nbsp; However, only a minimal effect was seen for response to the bitter tastant primaquine, or for response to copper or SDS ( Figure 1A ).&nbsp; These results mirror the previous finding that EGL-4 selectively regulated response to quinine and amodiaquine, but not primaquine, copper or SDS (Krzyzanowski et al., 2013), and further support a selective role for cGMP in regulating response to distinct stimuli.&nbsp; While it is not clear why cGMP signaling has only a minimal effect on primaquine response, one possibility is that this bitter tastant may signal through an aversive sensory neuron(s) other than ASH.

To determine whether multiple PDEs might work together to regulate quinine and amodiaquine response, we tested individual loss-of-function mutations in *pde* genes.&nbsp;While the quadruple mutant animals lacking function of the four PDEs were defective in response to both bitter tastants, single mutants all responded similarly to wild-type animals ( Figure 1B and 1C).&nbsp;This suggests that a combination of PDEs, possibly functioning in different neurons within the modulatory circuit (Chaubey et al., 2023; Krzyzanowski et al., 2016; Sojka et al., 2025), coordinate to regulate cGMP levels and ASH sensitivity to quinine and amodiaquine.&nbsp;According to transcriptional profiling data reported by the *C. elegans* Neuronal Gene Expression Network (CeNGEN, https://cengen.shinyapps.io/CengenApp/), each is expressed in multiple neurons within the circuit ( *pde-1* : AWB, AWC, AFD, RMG; *pde-2* : AWB, ASK, AWC, RMG, ASH, AFD, ADL, ASJ, ADF, ASI, AIA; *pde-3* : RMG, AWC, AFD, AWB, ADF, ASK, ASI, ASH; *pde-5* : AFD, AWC, ASI, ASJ, ASK, AWC), in addition to other cells (Hammarlund et al., 2018). Future experiments will be directed at testing each double and triple *pde* mutant combination, along with cell-specific rescue experiments, to identify the subset of PDEs that regulate bitter taste avoidance and their site of action.&nbsp;Taken together, our work suggests that both cGMP generation and breakdown contribute to modulating animal sensitivity to noxious stimuli and highlights a previously unknown role for PDEs in this process.

## Methods

Strains were maintained under standard conditions on NGM agar plates seeded with OP50 *Escherichia coli* bacteria (Brenner, 1974).&nbsp; Behavioral assays were performed as previously described (Chaubey et al., 2023; Ezak et al., 2010; Fukuto et al., 2004; Krzyzanowski et al., 2013; Krzyzanowski et al., 2016; Polk et al., 2025; Sojka et al., 2025). Well-fed young adult *C. elegans* were tested 20 minutes after transfer to NGM plates lacking bacteria (“off food”).&nbsp; Responses to soluble taste stimuli were scored as the percentage of animals that initiated backward locomotion within four seconds of entering the drop of stimulus placed in front of a forward moving animal. Each animal was tested only once.&nbsp; Each plate of 10 animals counted as one replicate, performed in duplicate each day, over at least three separate days in parallel with controls. Tastants were dissolved in M13 buffer, pH 7.4 (Wood, 1988). The Student's two-tailed t-Test ( Figure 1A ) and one-way Anova with Dunnett's multiple comparisons test ( Figure 1B and 1C) were used for statistical analyses.&nbsp; In the figure * denotes p < 0.05, **** denotes p < 0.0001, and ns denotes p ≥ 0.05.

**Table:** 

**Strain**	**Genotype**	**Source**
N2	Laboratory wild-type	CGC
TQ1828	*pde-1 ( nj57 ) pde-5 ( nj49 )* I; *pde-3 ( nj59 )* II; *pde-2 ( tm3098 )* III	CGC (Liu et al., 2010)
TQ1116	*pde-1 ( nj57 ) * Allele: deletion in exons 5 and 6, frame shift	Gift of Ikue Mori and Shawn Xu (Liu et al., 2010)
FX3098	*pde-2 ( tm3098 )* 0x outcross Allele: deletion of part of exon 3, frame shift	NBRP (Liu et al., 2010)
FG1092	*pde-2 ( tm3098 )* 5x outcross	This study
TQ1118	*pde-3 ( nj59 ) * Allele: deletion in exons 3 and 4, frame shift	Gift of Ikue Mori and Shawn Xu (Liu et al., 2010)
TQ1120	*pde-5 ( nj49 ) * Allele: deletion spanning exons 4–9	Gift of Ikue Mori and Shawn Xu (Liu et al., 2010)

## Reagents

Quinine hydrochloride dihydrate (Q1125-10G), amodiaquin dihydrochloride (A2799-5G), primaquine diphosphate (160393-1G), and copper (II) chloride hydrate (C3279-100G) were purchased from Sigma-Aldrich.&nbsp; SDS (BP166-500) was purchased from Fischer Scientific.&nbsp; CGC = *Caenorhabditis* Genetics Center. &nbsp;NBRP = National Bioresource Project for the Nematode (Mitani lab, Tokyo Women's Medical University School of Medicine, Tokyo, Japan).
